# Absinthism: a fictitious 19th century syndrome with present impact

**DOI:** 10.1186/1747-597X-1-14

**Published:** 2006-05-10

**Authors:** Stephan A Padosch, Dirk W Lachenmeier, Lars U Kröner

**Affiliations:** 1 Universitätsklinikum Heidelberg, Klinik für Anästhesiologie, Im Neuenheimer Feld 110, D-69120 Heidelberg, Germany; 2 Chemisches und Veterinäruntersuchungsamt (CVUA) Karlsruhe, Weißenburger Str. 3, D-76187 Karlsruhe, Germany; 3 Institut für Rechtsmedizin der Universität zu Köln, Melatengürtel 60–62, D-50823 Köln, Germany

## Abstract

Absinthe, a bitter spirit containing wormwood (*Artemisia absinthium *L.), was banned at the beginning of the 20^th ^century as consequence of its supposed unique adverse effects. After nearly century-long prohibition, absinthe has seen a resurgence after recent de-restriction in many European countries. This review provides information on the history of absinthe and one of its constituent, thujone. Medical and toxicological aspects experienced and discovered before the prohibition of absinthe are discussed in detail, along with their impact on the current situation. The only consistent conclusion that can be drawn from those 19^th ^century studies about absinthism is that wormwood oil but not absinthe is a potent agent to cause seizures. Neither can it be concluded that the beverage itself was epileptogenic nor that the so-called absinthism can exactly be distinguished as a distinct syndrome from chronic alcoholism.

The theory of a previous gross overestimation of the thujone content of absinthe may have been verified by a number of independent studies. Based on the current available evidence, thujone concentrations of both pre-ban and modern absinthes may not have been able to cause detrimental health effects other than those encountered in common alcoholism. Today, a questionable tendency of absinthe manufacturers can be ascertained that use the ancient theories of absinthism as a targeted marketing strategy to bring absinthe into the spheres of a legal drug-of-abuse. Misleading advertisements of aphrodisiac or psychotropic effects of absinthe try to re-establish absinthe's former reputation. In distinction from commercially manufactured absinthes with limited thujone content, a health risk to consumers is the uncontrolled trade of potentially unsafe herbal products such as absinthe essences that are readily available over the internet.

## Introduction

**Figure 1:**
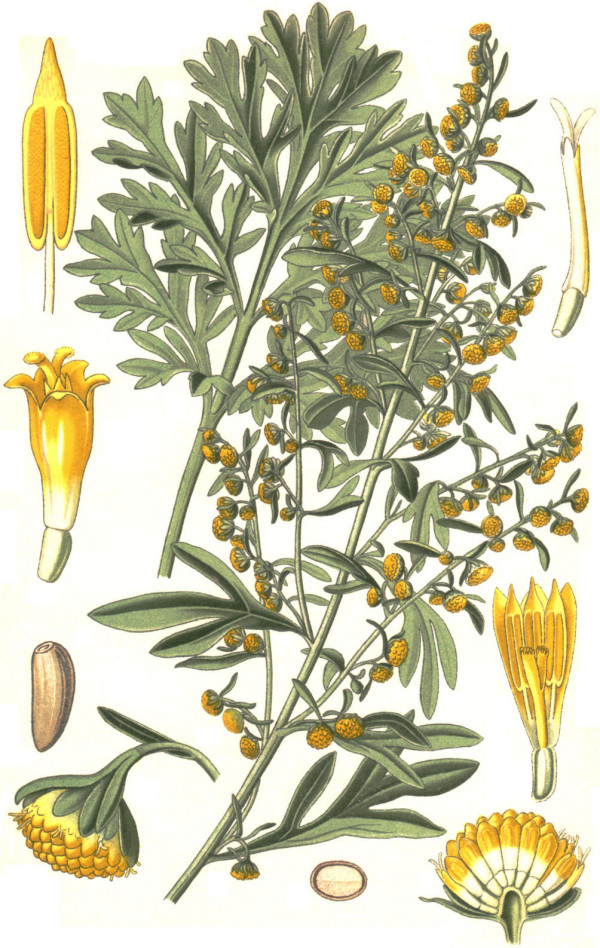
Wormwood, *Artemisia absinthium *L., drawing of plant, flowers, seeds and fruits (drawing by W. Müller, 1885 reproduced from Thomé [106]). Wormwood is the characteristic aromatic component of absinthe.

Absinthe – a bitter spirit containing wormwood (*Artemisia absinthium *L.) (Figure 1) and other herbs – was one of the most popular alcoholic beverages of late 19^th ^century Europe. The emerald green drink was consumed by people from all walks of life, including the bohemian upper class, artists, poets and intellectuals. While the lower classes celebrated *l'heure verte *(the green hour) in numerous bars and cafés, painters and poets created famous paintings and poems dedicated to the "green fairy." Absinthe was popular in *fin-de-siècle *Paris and *la vie bohème *of Prague. The most remarkable celebrity known as an absinthe drinker is the Dutch post-impressionist painter Vincent van Gogh (1853–1890, Figure 2), whose illness is still a matter of debate among neurologists and psychiatrists [1-7]. Other famous painters of the time, such as Henri de Toulouse-Lautrec and Paul Gaugin, and illustrious poets like Oscar Wilde, Charles Baudelaire, and Edgar Allan Poe were all fond of absinthe.

**Figure 2:**
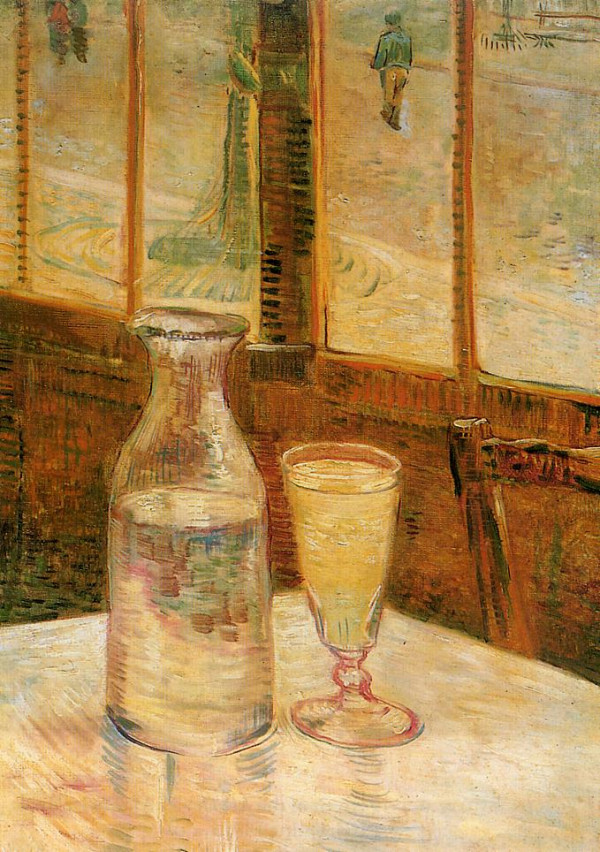
Vincent van Gogh: Still Life with absinthe (Paris 1887). The picture shows one of the countless cafés in Paris, in which absinthe was served. Next to the glass filled with absinthe, a water bottle is illustrated, which was necessary for drinking ritual.

**Figure 3:**
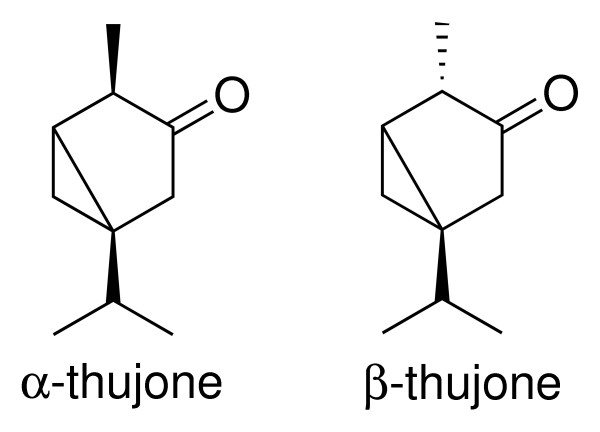
Structure of α- und β-thujone, the principal components of wormwood oil (*Artemisia absinthium *L.).

Because absinthe consumption reached excessive and alarming proportions at the turn of the 19^th ^century, many European governments, as well as the U.S. administration, successively banned the icon of *la vie bohème *by several prohibition acts. Absinthe was used as an easy target of the temperance movement with the aim of later prohibiting alcohol in general. But absinthe remained a singularity as the only kind of alcoholic beverage with a long-term ban. In some European countries (e.g. UK, Spain, Czech Republic), however, the "green fairy" survived, but consumption was comparatively low. The European Council enacted in 1988 the directive "on the approximation of the laws of the Member States relating to flavorings for use in foodstuffs and to source materials for their production," that re-allowed wormwood as ingredient of alcoholic beverages. However, maximum limits of the wormwood ingredient thujone (Figure 3), which was speculated to be the most probable cause for absinthism, were issued [8].

Because of this change of policy, absinthe has seen a recent resurgence. In contrast to the social, legal and medical problems of the late 19^th ^century, today the image of the "green fairy" has markedly changed, but still remains titillating. Today's so called "new absinthe" is offered as a newly fashionable, exclusive drink for yuppie parties with claimed properties ranging from spiritual elucidation to aphrodisiac stimulation – with corresponding pricing. In parallel, a fan club within the internet community has emerged. Absinthe can be purchased via the internet from various countries worldwide, making it possible to receive it in countries where it is not legally available. Moreover, numerous recipes for the self-production of absinthe are available on the internet. To date, it is unclear if the re-licensing of absinthe will cause similar or even new and different forensic, medical and social problems as it did in the late 1800's.

This article provides information on the history of absinthe and a prime constituent, thujone. Medical and toxicological aspects experienced and discovered before the prohibition of absinthe are discussed in detail, along with their impact on the current situation. It is the intention of the authors to provide a comprehensive overview of this topic of multi-faceted interest and to discuss this issue objectively.

## The rise and fall of wormwood spirits

Documented medical use of wormwood can be dated back to the Ebers Papyrus, an Egyptian medical document dating from about 1552 B.C. and the oldest preserved medical document [9]. This papyrus is believed to be a copy of the even more ancient books of Thoth (3500 B.C.). The name "wormwood" is derived from its anthelmintic properties, which were recognized by the ancient Egyptians.

Wormwood, in the context of its bitter taste, is mentioned several times in the Old Testament (Jeremiah 9:15, 13:15). In the biblical context, the plant represented a curse, calamity (Lamentations 3:15) or injustice (Amos 5:7). In Revelations 8:11, the Greek equivalent *ho apsinthos *is used as a name for a star that fell into the waters and turned them bitter. The Greek word *apsinthion *– undrinkable – is most probably the ancestor of the word absinthe. The Greek mathematician and philosopher, Pythagoras of Samos (569-475 B.C.), recommended wine-soaked wormwood leaves to alleviate labor pains; Hippocrates (~460-377 B.C.) used wormwood extracts for the treatment of menstrual pain and rheumatism [10].

Pliny the Elder (23–79), the Roman scholar and scientist, also mentioned extracts of wormwood in his opus *Historia Naturalis *[11]. In the Middle Ages, wormwood was used as a purge and vermifuge, and it developed towards a "general remedy for all diseases" and was "a herb of Mars" for its medical powers [10]. Wormwood's bitter taste inspired women in those days to apply it to their nipples to encourage the weaning of their babies. In fact, Shakespeare has Juliet's nurse expound upon this in *Romeo and Juliet*.

The image of just a bitter medicine changed to a popular drink among the masses in the 16^th ^century. The so-called *Purl *of Tudor England was a drink composed of hot ale and wormwood. Dried leaves of wormwood were infused in proof-spirits, distilled, and sweetened with sugar as prescribed in Smith's *Complete Body of Distilling *in 1731 [12]. The French physician Pierre Ordinaire is supposedly the originator of the classic absinthe recipe. Being acquainted with the ancient use of wormwood, he began to develop a recipe for an alcoholic drink, which probably contained wormwood, anise, hyssop, dittany, sweet flag, melissa and varying amounts of coriander, veronica, chamomile, parsley and (allegedly) spinach. Dr. Ordinaire, who had fled the French revolution, settled down in Val-de-Travers in western Switzerland, which has remained an important centre of absinthe production. In the small town of Couvet, the elixir (68%vol) soon attained the nickname *fée verte*.

After Dr. Ordinaire's death, his recipe came into the possession of Henri-Louis Pernod, who began the commercial production of absinthe in 1797. In 1805, Pernod moved to Pontarlier, France, to serve the French market; the distillery had one still with a daily capacity of 16 litres. The widespread use of alcoholic drinks containing wormwood extract might have also resulted from the use of wormwood as a preventive measure for helminthiasis and fevers during the French conquest of Algeria (1830–1847). The soldiers returning to France discovered absinthe as a tasty substitute for their wormwood medicine [13].

Due to a rising interest in anise-based spirits as well as increased promotion and advertising, the production of Pernod's absinthe was increased up to a 125,000 liter scale in 1896. This was aided by the drastically reduced production of red wine in these years due to major damages caused by the vine pest. The emerald spirit was, however, known to be enjoyed excessively on both sides of the Atlantic [14].

The annual per capita consumption of absinthe in France increased fifteen-fold between 1875 and 1913. According to an article in The Times (1915), French consumption of pure alcohol in 1876 was 15,500 hectoliters; it was 10 times that amount in 1908, and in 1913 it had reached the figure of 239,492 hectoliters, representing 60 liters per inhabitant [15]. Parallel to this mass consumption and its consequences, anti-alcohol movements, winegrowers and clergy called for the banning of absinthe. Many murders and other acts of violence were attributed to the influence of absinthe.

Furthermore, the medical community had developed a strong scientific and medical case against absinthe, attributing an increase in insanity and other serious medical problems to an overindulgence in the drink [13]. It was widely believed that the problem with alcohol was not the quantity consumed but the quality. The absinthe prohibition crusade in France was a paradoxical campaign in which the wine-producers, suppliers of the vast majority of alcoholic drinks consumed, backed the temperance movement [16]. The attention being given to absinthe's supposed unique qualities can be seen as an attempt to reduce alcoholism without specifically touching alcohol. However, it also may have diverted efforts away from the genuine dangers of heavy alcohol consumption [16].

At first, concerns about absinthe were ignored, especially by the French government, due to lucrative revenues resulting from the enormous scale of absinthe sales. By the end of the 19^th ^century, temperance forces had succeeded in getting the attention of almost all of France through educational programs and public awareness campaigns. In 1908 a bill was passed that, ironically, increased the amount of alcohol in absinthe, the argument being that the requirement for higher alcoholic strength would eliminate those producers who used artificial essences with lower standards of purity [13]. Only rising concerns about a weakening of military power in the light of absinthe abuse, especially in the army, pressured the French government to ban absinthe in 1915. The U.S. Department of Agriculture had already issued the Food Inspection Decision 147, which banned absinthe in the U.S., on 25^th ^July 1912. Belgium, Switzerland and Italy had also passed laws prohibiting absinthe in 1905, 1908 and 1913 respectively; finally, Germany outlawed the green fairy on 27^th ^April 1923 [17].

Prestwich concluded that the prohibition of absinthe did little to improve the health of the French people as deprived of their traditional absinthe consumers merely switched to similar drinks. In addition, by stressing the problem of essences and impure alcohol, temperance campaigners distracted both medical research and the public from the real cause of alcoholism, namely the excessive consumption of any type of alcoholic drink [18].

For further information about the social history of absinthe, which goes beyond the scope of this review, the book of Adams is recommended [16]. Further information is available in the works of Arnold [19,20], Baker [10], Conrad [21], Lanier [13], Marrus [22], and Prestwich [18]. Information about absinthes' paraphernalia and the drinking ritual is available in an article of Hood [23].

## Definition of pre-ban absinthe

The drink to which we refer as "pre-ban absinthe" was the icon of the *belle époque*. When dealing with good quality absinthes, recipe books distinguished between *absinthe Suisse*, with an alcohol content of approximately 68–72% vol, *absinthe demi-fine*, with 50–68% vol and *absinthe ordinaire*, with a content of 45–50% vol. *Absinthe suisse *was considered the highest quality and consisted of pure herbal distillate, while in the other types, the distillate was diluted with ethyl alcohol. According to these widely ranging contents, these absinthes must have contained different concentrations of thujone.

A definition of absinthe was provided in Swiss law at the time of the prohibition of absinthe. According to this definition, every spirit drink, without regard to its method of production, that contains aromatic compounds of wormwood herb in combination with other aromatic compounds derived from plants such as anise and fennel, is defined as absinthe [24]. Thujone was regarded as being the determining factor amongst the aromatic compounds in terms of detecting wormwood spirits [25].

In the first step of traditional recipes for distilled absinthe, wormwood and other dried herbs (e.g., anise, fennel) are macerated. The macerate of the wormwood herb is of greenish-brown color, smells aromatic, like all *Artemisia *species, and reminds one of the composite herbs, like camomile. The taste is lightly stinging, strongly bitter and camphoric. The following distillation of the macerate results in a distillate that is reduced from the bitter compounds, which are relatively non-volatile. The distillation is conducted in a still with a very flat helmet slowly heated in a water or steam bath to avoid boilover that would negatively influence the product quality [26,27]. The distillation process is usually stopped at an alcoholic strength of 60%vol [27,28]. The characteristic, lightly volatile, fine aromatic components of the wormwood aroma appear in the first fraction between 80 and 60% volume, while the middle fractions posses a cinnamon or clove-like aroma [29]. Distillation of absinthe should never be carried on to the end, as the taste of the product would be too strong, and less fine [26]. Therefore, only the main fractions (heart) are used for the production of high-quality absinthe. The heads and tailings are collected separately and added to subsequent macerations or used to make *absinthe ordinaire *after renewed rectification [28].

As emphasized by Arnold [20], the distillation of absinthe may have been a type of "steam distillation" as significant amounts of water were added to the alcoholic macerate prior to distillation. Due to the influence of steam distillation, higher thujone may have been distilled over [20].

In the second step, wormwood (usually *Artemisia pontica*) and other herbs are added to the colorless distillate. This is done to accomplish the characteristic green colouring by chlorophyll and to achieve a mild bitter taste, as well as to extract other aromatic compounds. Because of the easy denaturation of chlorophyll through light and warmth, the characteristic color of a traditionally produced absinthe is pale greenish-yellow. Afterwards, the beverage is diluted with water until drinking strength is reached.

Typical historic recipes are given in the books of Duplais [30], Fritsch [27], Bedel [31] and de Brevans [28]. The composition of herbs used along with the wormwood differs from recipe to recipe. To improve the taste or add coloring, anise, star anise, lemon balm, hyssop, juniper, nutmeg, veronica, angelica root, melissa, coriander, camomile or parsley were added. Each country produced its own types of absinthe. For example, in the Czech Republic, peppermint was added, but neither anise nor fennel. In Switzerland, melissa, hyssop or angelica root were added to the Swiss alpine wormwood, which was a valued ingredient due to its strong aroma [32], while in France, coriander was added.

Because the essential oils from the diverse herbs can be kept in solution only in high alcohol concentrations, the addition of water causes a precipitation visible as an opaque clouding of absinthe. This phenomenon is called the Louche effect. The characteristic bitterness is caused by sesquiterpene-lactone absinthin, which can still be organoleptically detected in a concentration as low as 1 g in about 70 liters. Due to different historical aspects of absinthe, a sub-division into the historic "pre-ban absinthe" and the currently available "modern absinthe" will be used in this article.

## Pre-ban absinthe – a target for food adulteration

Besides the above-mentioned herbal ingredients, different manufacturers of absinthe sometimes used strange or even toxic additives such as methanol, sweet flag (*Acorus calamus *L.), tansy (*Tanacetum vulgare *L.), nutmeg (*Myristica fragrans *HOUT), antimony, aniline green, copper sulfate and cupric acetate indigo.

The Lancet reported that, in the days of pre-ban absinthe, antimony (antimonyl tartrate) was added with the well-meant intent to decrease absinthe's toxicity. However, it was questionable even in 1873 if "quantities of tartar emetic" would not rather adulterate the spirit as it would cause nausea, sickness and toxic effects of its own [33]. From today's view it is more likely that antimony salts were added to make absinthe turn milky when adding water simulating the Louche effect. Increasing consumption, which arose competition among the manufacturers, flooded the market with such imitations of absinthe. Absinthe can so easily be adulterated that Emerson wondered if the genuine article was still in existence [34].

In addition, instead of traditional production by distillation, absinthe could be made using herbal essences. According to Tibbles, the color of properly made absinthe is entirely due to chlorophyll derived from the green leaves of wormwood, hyssop, spinach, parsley, nettles and veronica; however, in the years preceding 1912, the spirit was most frequently colored by artificial agents [35]. Convenience products like absinthe extracts, which had only to be dissolved in alcohol and colored with food dye [27], were also commercially available at that time. As food adulteration, the light green color of chlorophyll was sometimes enhanced with inorganic salts like copper sulphate or copper acetate [36]. Inferior and falsified products were typically made by mixing industrial alcohol with flavorings and artificial food dyes, in the worst case with antimony trichloride, and copper salts.

Another general problem at that time was that heads and tailings, which were separated from the product fractions during distillation by legal manufacturers, were purchased by illegal manufacturers and used as a main component for adulterated absinthe products. The alcohol employed for absinthe was described to have been "frequently very impure" [37]. Emerson also wondered if total abolishment had occurred if the beverage had remained in its purity [34]. Given these facts, it is easily comprehensible that the prohibition of such a mixture could successfully eradicate a whole syndrome overnight.

## Modern absinthe

Most absinthe brands available today contain mainly the same herbal ingredients and extracts as pre-ban absinthe. Absinthe produced within the European Union is limited in its thujone content to 35 mg/l (maximum limit for bitter spirits) [8].

Top grade absinthe products are still manufactured according to traditional recipes, without the addition of dye or other additives. Some products are made of herbal distillates and are differentiated by a mild flavor. Because such products are colorless, they are sold as *Blanche *or *La Bleue*. Types with a lower alcoholic strength and added sugar are sold as absinthe-liqueurs. Independent of traditional recipes, many products sold nowadays are made with readily bought finished extracts of wormwood or other plants, which are blended with ethyl alcohol of agricultural origin. For the coloring artificial dye is used, especially mixtures of tartrazin (E102, FD&C Yellow No. 5) and patent blue V (E131) or brilliant blue FCF (E133). Inferior products contain no herbal extracts and are made solely by the blending of artificial flavors, coloring and ethyl alcohol [17].

In cases like this, sometimes even the macerated herbs are not distilled but only filtrated, diluted to drinking strength and bottled. These products have a strong pronounced taste of wormwood and a very strong, bitter taste. Further falsification is possible with the addition of extracts of other thujone-containing plants (e.g., *Thuja occidentalis L., Salvia officinalis L*.).

## Nineteenth century studies about absinthism

### Clincial effects of absinthism

When discussing the clinical effects of thujone and absinthe, it should be kept in mind that the majority of the data available was derived from clinical observations made in the late 1800's and are therefore lacking reliability and clinical significance.

With the increasing mass consumption of absinthe, more and more of the chronic – and most probably high-dose – absinthe consumers developed seizures, speech impairment, sleep disorder, mental prostration, auditory and visual hallucinations and finally death. This collection of symptoms gave birth to the term "absinthism;" it is unclear, however, if this syndrome ever really existed at all. Absinthism in these days was supposedly further characterized by brain damage, gastrointestinal problems, risk of psychiatric disease and even suicide [38]. Even an increased incidence of oesophageal cancer in absinthe drinkers was noticed [39]. In contrast, other authors recommended moderate doses of absinthe as a valuable remedy against depressions [40].

Both the serious and the populist medical literature of the day demonized absinthe, in many cases laying the groundwork for the anti-absinthe temperance movement. The definition of absinthism as a particular syndrome separate from alcoholism is intimately connected with the French physician Valentin Magnan. A biography of Magnan is available in a recent review article [41]. In Magnan's work about absinthism between 1864 and 1874 he described visual and auditory hallucinations accompanied by alterations in consciousness after consumption of absinthe [42,43]. Other authors described acute symptoms of absinthe, such as hallucination, restlessness, confusion, delirium and seizures [44] (Table 1). Symptomatic differences between the drinker of absinthe and the ordinary alcoholic were presented at the First International Eugenics Congress: in absinthism, the "hallucination insanity" was described to be "more active with sudden attacks of delirium, more terrifying, sometimes provoking most dangerous reactions of extreme violence" [45]. In addition, complete statistics of the central service for the admission of insane persons for the town of Paris were given. In the years 1867–1912, a number of 16,532 patients were treated for alcoholic intoxication. 70.3% of all patients were diagnosed as "chronic alcoholics," but only 1.0% of all patients were found to have symptoms of absinthism. Due to the high consumption of absinthe in Paris of that time, one questions if very severe forms of chronic alcoholism were misleadingly described as absinthism.

**Table 1:** Main acute and chronic effects of absinthe reported in the 19^th ^century

**Acute effects**	**Chronic absinthism**
vertigo	mania
seizures	softening of the brain
nervous debility	general paralysis
hallucinatory delirium	psychosis

In a clinical report on a case of absinthe intoxication, published in the *Boston Medical and Surgical Journal *(today the *New England Journal of Medicine*) in 1868, Amory, a former pupil of Magnan, observed "pleural effusions", "epileptiform seizures" and a "reddish discoloration of the urine" [38]. The latter symptom can be interpreted – among other possible causes – as an episode of acute porphyria. It has been shown *in vitro *that thujone and other terpenes exhibit porphyrogenic properties. In primary chicken embryo liver cell cultures, an acute porphyria-like state was mimicked by the addition of the iron chelator desferrioxamine. Upon addition of thujone, a marked accumulation of protoporphyrin was observed. Bonkovsky et al. concluded that the tested terpenes, i.e., thujone, are porphyrinogenic and hazardous, especially to patients with underlying defects in hepatic heme synthesis [46].

### Animal experiments with wormwood extract

It has to be stressed again that clinical reports of these days were more or less only descriptive or speculative, as causal connections between absinthe and thujone and the above-mentioned symptoms could never reliably be proven. However, experimental studies were performed on animals to prove that wormwood was the causing agent of absinthism, especially the seizures. All these studies were carried out using so-called "essence d'absinthe" (pharmaceutical wormwood extracts) or pure etheric oil of wormwood, however, often only the term "absinthe" was used for these extracts. Even in current history books the terms "absinthe" and "essence d'absinthe" are misleadingly used as synonymous [13].

If injected in pure form, wormwood extracts and alcohol showed distinctly different symptoms (Table 2). These results were generalized and transferred to humans who drank high concentrations of alcohol in combination with low concentrations of wormwood extract. In further experiments, guinea pigs – among other small animals – were placed in the presence of wormwood essence, while the control guinea pig was "shut up with a saucer of pure alcohol." In contrast to the animal breathing alcohol, which was reported to have simply become drunk, the guinea pig inhaling the vapors of wormwood experienced initial excitement and subsequently seizures [43].

**Table 2:** Comparative table between symptoms of absinthism and alcoholism according to animal experiments of Amory (1868) [38].

**Absinthism (Injection of pure wormwood extract (0.8–4.5 g) into the stomach of different animals)**	**Alcoholism (Injection of alcohol (0.8–5 g) into the stomach of different animals)**
Animal perfectly well for fifteen minutes, at the least after the ingestion; with the exception of a few muscular twichings and a slight uneasiness.	In a very few minutes symptoms of inebriation resulting in torpor.
Musuclar agitation, commencing in the anterior portion of the body.	Paralysis, commencing in posterior extremities, and then extending to the anterior.
No paralysis.	Paralysis of both posterior and anterior extremities in succession.
Epileptiform convulsions and rigidity, resulting in a rapid death.	No convulsions. Stupor, coma, resolution and a gradual death.
No apparent lesion, except, perhaps a slight cerebral congestion, showing the cause of death to be intoxication of the poison.	Lesions of the brain and of the alimentary canal; gastritis and enteritis might have supervened, had the animals lived long enough for their development

These experiments and deductions of Magnan et al. were criticized as early as 1869, when *The Lancet *commented upon the inadequacy of the evidence produced in order to prove that absinthism was different in its nature from chronic alcoholism. The sleeplessness, the tremor, the hallucinations, the paralysis, and even the seizures, were described to be well-known symptoms of simple alcoholic excess. The effect of inhalation of concentrated wormwood fumes was seen as not transferable to the effect of very small, continuous oral doses [47]. In Great Britain, the hostility against Magnans experiments led so far that the Royal Society for the Prevention of Cruelty to Animals prosecuted three English doctors for assisting Magnan in 1874 in demonstrating that intravenous injection of wormwood extracts into a dog induced epilepsy. The prosecution failed as Magnan had discreetly returned to France [48].

Further investigations were undertaken to determine the origin of the fits in wormwood epilepsy by Boyce [49]. For this purpose, numerous lesions were made both in the brain and spinal cord of cats, and wormwood administered immediately or after a lapse of days or weeks. It was found that wormwood, acting upon the bulbo-spinal centers (including the cerebellum) alone, could produce a series of "clonic fits", differing from the cortical in the slower rhythm of the contractions. In experiments of Ott upon rabbits those results were disputed as no "spinal" but only "cortical convulsions" were determined, which raises the question if the crude techniques of the 19^th ^century were suitable at all to determine the physiological origin of epilepsy [50].

In the noteworthy work of Ossipow, the problem of different wormwood extracts to achieve epilepsy in animals was discussed for the first time [51]. The failure of some researchers to replicate the experiments of Magnan was explained by misunderstandings between "absinthe", "essence d'absinthe", and "extrait d'absinthe". Ossipow stressed that only the "essence d'absinthe" (alcohol-free wormwood oil) and not the ready-to-drink alcoholic beverage (in France called "extrait d'absinthe") is usable to trigger seizures in animals.

Further studies were conducted by Cunningham [52] and Lesieur [53]. The only consistent conclusion that can be drawn from all these animal experiments is that wormwood oil but not absinthe is a potent agent to cause seizures in animals.

### Degeneration and absinthism

A further strong argument of the anti-absinthe phalanx was grounded on the Lamarckian theories of the inheritance of diseases (Jean-Baptiste Lamarck, 1744–1829). According to these theories, any traits acquired by absinthe drinkers would be passed on to their children. The idea of degeneration was also used by Magnan to explain mental illnesses [54]. It is interesting to note that this hereditary feature was also ascribed to alcoholism. The editorial section of JAMA published the theory that a larger proportion of the children of alcoholics were more "idiotic, epileptic, neurotic, alcoholic, degenerate and deformed" than the children of healthy parentage, and total abstinence was postulated [55].

The condition of absinthism was introduced into late 19^th ^century medicine together with the first emerging descriptions of alcoholism [37]. Intriguingly, this fact could hold the key for the solution of the debate about whether absinthism was a clinical pattern of its own and how it should be distinguished from chronic alcoholism. As mentioned previously, due to the low solubility of etheric oils, absinthe usually contains high concentrations of ethanol, which means that there was no ingestion of thujone without ingestion of remarkably high quantities of ethanol.

Recently, in an editorial, Strang et al. raised the question of "absinthe: what's your poison?" [56]. To us, however, the question is really what happened to the symptoms of absinthism after its prohibition. Did this mysterious syndrome disappear abruptly or did these symptoms simply continue to exist among chronic alcohol abusers under the name of alcoholism, which seems to be more tolerated by society? Finally, as with so many facets of the green fairy, this issue remains controversial and perhaps will never be solved.

## Modern studies about pharmacology and toxicology of thujone

In the 20^th ^century, as a consequence of the description of the bicyclic monoterpene thujone as the main component of wormwood oil, the main focus of scientific studies was changed from the research of wormwood extract to isolated thujone. It must be stressed, however, that besides the β-thujone chemotype of the wormwood plant further chemotypes were described, which contain *cis*-chrysanthenylacetat, *cis*-chrysanthenol, *cis*-expoxyocimene, sabinylacetate or bornylacetate as principal component [57-63]. In the west alpine area above 1000 m the *cis*-epoxyocimen type is predominat, while the β-thujone type rather exists in the lower zones [60]. In wormwood oil from the Tuscany [64] or the Pyrenees [58] neither α- nor β-thujone could be detected. These significant differences in composition of wormwood may also be attributable for the previously described failure of some researchers to replicate the animal experiments of Magnan.

The acute and chronic toxicology of thujone were reviewed in the WHO Food Additives Series 16 [65] and more recently by the Scientific Committee on Food of the European Commission [66]. The principal data are summarized in Table 3. The toxicological evaluations led to the establishment of maximum limits for thujone (35 mg/l in bitters) by the Codex Alimentarius Commission of the FAO/WHO [67], which were adopted by many countries including the European Union [8] and Switzerland [68] but not the USA, where manufacture and importation of absinthe is still prohibited [69,70]. It was noted, however, that in the USA consumption and possession remained legal, so that travelers returning to the USA with a bottle or customers buying it from Europe on the internet are not guilty of any crime, though they could have their bottle confiscated [71].

**Table 3:** Summary of data about toxicology of thujone

Toxicity data		Reference
oral LD_50 _in rats	192 mg/kg bw	[66]
oral LD_50 _in rats	500 mg/kg bw	[107]
iv LD_50 _in rabbits	0.031 mg/kg bw	[107]
NOEL for convulsions in rats	12.5 mg/kg bw (males)	[108]
NOEL for convulsions in rats	5 mg/kg bw (females), 10 mg/kg bw (males)	[66]
NOEL for convulsions in rats	5 mg/kg bw	[66]
TDI (based on NOEL with safety factor of 500)	10 μg/kg bw/d	[66]
Metabolism	2-,4-, and 7-hydroxylation	[75,76]
Mechanism of toxicity	GABA Type A modulation (α-thujone neurotoxicity, convulsant effects)	[72,74,76]
Mechanism of toxicity	Porphyrogenicity (determined in cultures of chick embryo liver cells)	[46]
Behavioral effects	5-HT_3 _receptor modulation, but no conclusive evidence for psychotropic actions of thujone	[77]

Until today, only little valid data are available concerning the effect of α-/β-thujone, especially in regard to the influence on the central nervous system after absinthe consumption. In comparison to β-thujone, α-thujone is believed to be 2.3 fold more toxic [72]. A recent study of Dettling et al. showed that the administration of alcohol containing a high concentration of thujone (100 mg/l) had a negative effect on attention performance [73]. When the subjects were under the influence of alcohol or were administered both alcohol and low thujone concentrations (10 mg/l), these effects were not observed. Similarly, it was found that only high concentrations of thujone could temporarily counteract the anxiolytic effect of alcohol.

The interaction of α-thujone with γ-amino butyric acid (GABA) dependent chloride channels can explain its convulsant effects [72,74-76]. It was determined that α-thujone acts like many naturally occurring and synthetic convulsive agents (e.g. picrotoxin) by blocking GABA mediated inhibition. The effect on the brain is excitatory (analeptic). Anxiogenic and possibly alerting effects of GABA antagonists were also noted. However, Olsen commented that in absinthe one is balancing the effect of thujone with the intoxicating, disinhibitory, and depressant effects of ethanol [74].

Deiml et al. were not addressing the toxicity but instead were researching the 5-HT_3 _receptor as a potential site of psychotropic actions of α-thujone. In homomeric receptors, α-thujone enhanced the inherent channel-blocking potency of the natural ligand, 5-HT. In heteromeric receptors, α-thujone recruited an additional channel-blocking component of the agonist. The authors could, therefore, prove a reduction of the 5-HT_3 _receptor activity, but it stayed open if this inhibitory action on serotonergic responses contributes to behavioral effects of thujone [77].

Interestingly, the activation of human bitter taste receptors by α-thujone was recently proven by Behrens et al. and it was found that the receptor is sufficiently sensitive to serve as protection against the ingestion of toxic amounts of this substance [78]. However, it is questionable if these findings can be transferred to the ingestion of thujone in alcoholic beverages. Possible receptor interactions between thujone and ethanol as well as differences between sober and inebriated persons must be taken into account.

The sometimes observed porphyrinogenic effect of thujone and other terpenoids is explained with the pathway of metabolization by the hepatic cytochrome P-450 system [46,76,79]. Under the presumption of relatively high thujone concentrations of 260 mg/l, Bonkovsky et al. speculated that if there is an appreciable hepatic first-pass extraction and if the rate of hepatic metabolism is not unusually rapid, the concentrations in the livers of absinthe drinkers could have been in the 20–200 μM range. Such concentrations would be sufficient to produce porphyric crises in patients with underlying defects in hepatic heme synthesis. An additional effect of ethanol, perhaps acting synergistically, was also anticipated, since ethanol and other short-chain alcohols found in alcoholic beverages are porphyrogenic [46].

Intoxication due to wormwood or thujone rarely occurs, either due to a misconceived belief in folk remedies or simple ignorance [80]. In 1862, Smith described a case of ingestion of about 14 ml of oil of wormwood by a male adult. The patient was insensible, convulsed, the jaw clenched, and foaming at the mouth; tendency to vomit was also present [81]. To our knowledge, there is only one recent clinical case report by Weisbord et al. from the U.S. dealing with obvious acute thujone intoxication [82]. A 31-year-old male had ingested "herbal oil," which he had assumed to be the spirit absinthe and had purchased over the internet from a website that sold essential oils for aromatherapy. Several hours later, the patient became listless, suffered tonic-clonic seizures and finally developed rhabdomyolysis and then acute renal failure. It is tempting to speculate that these symptoms were caused by thujone, however other ingredients of the herbal oil cannot be excluded as the culprit.

Very few data published only in non peer-reviewed literature exist about the pharmacology of thujone. Max pointed out that the typical 2–4 mg of thujone, which were consumed per drink were far below the level at which acute pharmacological effects are observed [83]. This is confirmed by Hinkelbein, who states that by the consumption of absinthe, up to a blood alcohol concentration of 2.5 g/l, approximately 3.5 mg of thujone are ingested (0.005 mg/kg bodyweight) [84]. In this order of magnitude, it is highly improbable that central effects can be caused by thujone.

A pilot drinking study by Kröner et al. resulted in high blood alcohol concentration, but as expected no thujone was detected [85]. The probands examined did not show any central effect caused by the terpenoids besides the effect of the alcohol. Therefore, the adverse potency of absinthe can be neglected, if the EU limit is obeyed.

The German federal institute for risk assessment holds the view that, even if the legal limit of 35 mg/l is significantly exceeded, the consumer does not ingest health-threatening amounts of thujone. Because of the high alcoholic strength it is advised against a continuous and excessive consumption [86].

## Toxicological rehabilitation of absinthe

Until recently, the thujone content of pre-ban absinthe was largely unknown and was calculated in 1992 by Arnold [20] to be as high as 260 mg/l (a value very often cited in the newer literature, e.g. Refs. [10,16,46,56,87,88]). The value of 260 mg/l was determined on the basis that 100 l of absinthe employed 2.5 kg of dried *Artemisia absinthium *(1.5% oil, of which 67% is thujone; corresponding to 251 mg/l of thujone in the final product) and 1 kg of dried *Artemisia pontica *for coloration (0.34% oil, of which 25% is thujone, corresponding to 9 mg/l of thujone in the final product) [20]. Max independently calculated a similar concentration [83]. These calculations assumed that the total amount of thujone would be recovered in the final product. The following three points were given by Arnold to support his calculation of relatively high concentrations. First, by adding water to the first decoction before heating, a type of "steam-distillation" was achieved wherein the amount of any constituent distilled over depends on both its vapor pressure and molecular weight. In this way the effect of a low vapor pressure for a particular compound may be counteracted to some extent by its high molecular weight relative to that of water. Second, the distillation head of the industrial apparatus was simple and little attempt was made to restrict carry-over by aerosol entrainment. And third, the purpose of the secondary extraction at moderate temperature was twofold, to achieve a green coloration and to add additional flavor [20].

However, it cannot be totally disregarded that during distillation a discrimination of thujone occurs. Historic recipe books prescribed the removal of the heads and tailings [26-28]. Duplais, for example, indicates that after maceration in 16 l of alcohol (85%vol) and addition of 15 l of water, only 15 l of product are withdrawn. 1 l of alcohol is discarded in the process [30]. In a non peer-reviewed magazine article, Turner described first experiments conducted by T. Breaux on a French distillery built in 1834. After distillation in a historic still built for absinthe, the thujone originally present in the macerate was not recovered in the distillate [71]. The thujone content of absinthe can then only be caused by the second coloration step, which would lead to a concentration of 9 mg/l according to Arnold's calculation.

Baker reports another calculation that resulted in thujone concentrations of 60–90 mg/l [10]. Wilson [89] estimated in 1936 that absinthe made from essences contained 1.8 to 45 mg/l, and absinthe made with wormwood contained 2 to 34 mg/l of thujone.

Hutton pointed out that the thujone content of pre-ban absinthes could have been overestimated because of the insufficient analytical methods that were available at the time [88]. Historically applied methods for the determination of levels of thujone in absinthe were based upon iodometric titration [90] or color reactions [91]; these sometimes provided only detection limits as high as 20 mg/l and were therefore unfit for the detection of small quantities. At the beginning of the 19^th ^century, the most modern methods were based upon the reaction of thujone with sodium nitroprusside, sodium hydroxide and acetic acid and provided a limit of detection of 5 mg/l [92]. However, this color reaction was highly unspecific and therefore other essential oils, aldehydes and ketones led to a similar reaction to thujone. Even by improved sample preparation, it was not possible to avoid these interferences. A positive reaction in the case of thujone analysis could not automatically be interpreted in such a way as to prove that the spirit in question was made with wormwood. However, a negative result was regarded as proof of the absence of wormwood oil [93].

The sensitive and selective determination of thujone in spirits was only possible by using modern chromatographic methods. The first gas chromatographic method with a flame ionization detector for the determination of thujone in alcoholic beverages was developed by Mérat et al. in 1976 [94]. In a recent study of our working group [95] absinthes produced according to historic recipes did only contain relatively low concentrations of thujone (mean: 1.3 ± 1.6 mg/l, range: 0–4.3 mg/l), which is 50–100 times below the NOEL (No observed effects level) of thujone for convulsions determined in animal experiments. A vintage absinthe from Tarragona (1930) showed a relatively low thujone concentration of 1.3 mg/l. Swiss Val-de-Travers absinthes from traditional small distilleries contained 9.4 and 1.7 mg/l of thujone. Krumm et al. verified our results by their production of absinthes after authentic French recipes. All manufactured products had thujone concentrations below 1.5 mg/l [96]. Hutton found 6 mg/l of thujone in a Pernod absinthe from 1900 [88]. In a non peer-reviewed magazine article, Ashcraft reports tests on pre-ban absinthes conducted by T. Breaux, who found thujone amounts around 5 mg/l [97]. Schaefer et al. found such low thujone concentrations (<0.01 mg/l) in a legal French absinthe dating from 1904 that the authors even proposed the "toxicological rehabilitation" of absinthe [98]. In a current study of the neuropsychiatric toxicity of absinthe by Luauté et al. it was concluded that recent toxicological studies do not prove, any more than in Magnan's time, that the beverage itself was epileptogenic [99]. The toxicity of pre-ban absinthes, as that of modern ones, was found to be essentially due to their alcohol content.

The theory of a gross overestimation of the thujone content was, therefore, verified by six independent studies [88,95-99]. The discrepancy between the experimental findings of pre-ban absinthes (low thujone concentrations) and the calculations of Arnold and Max (high thujone concentrations) [20,83] could not be resolved so far. Further research is needed to study the behavior of thujone during distillation. Considerable differences to the composition of the distillate may result between batchwise distillation of diluted alcohol and fractional distillation of an undiluted macerate.

Currently no experimental evidence does suggest that historic absinthes had such high thujone contents to cause toxic effects. On the contrary, the analyzed historic products appear to have complied with today's maximum limits derived to exclude toxic or other unwanted effects. The feared return of absinthism, proclaimed e.g. by Hein et al. [100], Holstege et al. [44] or Müller [101] is therefore exaggerated. The effects of the recent types of absinthe are predominantly caused by the naturally high alcoholic strength (>50%vol), although it is possible to reach effective thujone blood levels, if illegally produced and distributed absinthe is ingested.

## Present impact of absinthe due to change of policy in the European Union

The policy change in the European Union was primarily based on the fact that absinthe was never prohibited in some European countries including United Kingdom and Spain. Under regard of the toxicological studies given above, the prohibition in other European countries was seen as a trade barrier, so that a harmonization of the European law was enacted [8]. Even if a renewal of absinthism can be ruled out, the recent re-emergence of absinthe led to some new problems.

In a recent study it was noted that thujone concentrations of more than 10 mg/l were found in 22% of commercial samples [102]. Some of today's commercial samples appear to have higher thujone concentrations than pre-ban absinthes. This may be due to the questionable tendency of some absinthe manufacturers and suppliers to advertise the thujone content and supposed psychoactive or aphrodisiac properties of their products on their websites. The ancient theories of Magnan et al. are used as a targeted marketing strategy to bring absinthe into the sphere of a legal drug-of-abuse.

Baker alluded some of the anecdotal reports on the power of absinthe, which are detailed in a number of internet forums, to a mere placebo effect, especially since the brand in question contained virtually no wormwood at all [10]. A placebo effect is also a possible interpretation for the "vaunted aphrodisiac powers" of absinthe that were advertised in a 1971 Playboy feature on absinthe by Zolotow [103]. We found no scientific evidence supporting the conclusion from the article that "absinthe is one of the best and safest aphrodisiacs ever invented by the mind of man". However, the aphrodisiac effects are nowadays even promised on some absinthe bottle labels.

In addition, slogans such as "contains the maximum allowed thujone concentration of 35 mg/l" should be critically judged by the appropriate authorities. Some so-called absinthe essences (with high thujone contents of 750 mg/l) are even sold on the internet as a possible means for customers to "regulate the thujone content" themselves, so that "it is no problem anymore to step behind the 35-mg border." Absinthe is also often misleadingly advertised as having a cannabis-like effect. This is based on a hypothesis – again – from Magnan that absinthe acts in the same way as hashish [43]. The hypothesis was renewed in 1975 by relatively far-fetched findings stating that, because of structural similarities between thujone and tetrahydrocannabinol (THC), both substances might activate the same receptor in the central nervous system [104]; this could not be proven in later experiments by Meschler and Howlett [87]. The THC-absinthe connection may serve as an archetype of how conjectural scientific evidence can enter our modern culture. A search on Google for "absinthe and THC" produces approximately 36,400 hits mostly of shopping sites, which advertise absinthe for psychoactive effects. In one case, the declaration "cannabis-like effect" was even found on a bottle label.

In closing it should not remain unmentioned that some high-quality distilleries have re-created absinthes according to pre-ban recipes [97]. Hopefully, after the recent de-restriction of absinthe in Switzerland, absinthe's country of origin, further high-grade products may show up on the market. Switzerland also proposed to introduce protected geographic denominations of origin and protected geographic indications on the labeling of absinthe, as well as the ban of artificial food dyes.

## Conclusion

From this critical review of the literature, it is concluded that chronic abuse of absinthe did not cause any distinct syndrome. The so-called absinthism cannot exactly be distinguished from chronic alcoholism. The literature gives proof that the thujone concentrations of pre-ban absinthes were not able to cause such toxic effects (e.g. seizures) that were found in animal experiments with pure wormwood extracts. However, much of the literature is focused on thujone as the potentially toxic component of absinthe. The possibility remains that other constitutents found within wormwood or other ingredients of absinthe may cause potential health problems. The paucity of good scientific studies about absinthe, especially in the realm of chronic human consumption and long term effects of thujone-containing beverages must again be pointed out.

Based on the current available evidence, commercially manufactured absinthe appears to not cause detrimental health effects other than those encountered in common alcoholism. The exceptionally high alcoholic strength of absinthe (>50%vol) alone may lead to major health and social problems, but is not unique to this spirit. However, misleading advertisements of aphrodisiac or psychotropic effects of absinthe try to re-establish absinthe's former reputation. A health risk to consumers is also the uncontrolled trade of potentially unsafe herbal products such as absinthe essences that are readily available over the internet.

On absinthe, Marie Corelli once said: "Let me be mad, mad with the madness of absinthe, the wildest, most luxurious madness in the world" [105]. After having been banned from most European countries for almost a century, the emerald green, mysterious drink has returned to the market, resuming all the myths and legends of former years. After the green fairy had inspired the artistic and literary set of the *belle époque *and at the same time supposedly poisoned numerous people, the impact that absinthe will exert on modern society remains unclear.

## Competing interests

The author(s) declare that they have no competing interests.

## Authors' contributions

SAP and LUK were responsible for the original concept and design of the article and drafted the sections "The rise and fall of wormwood spirits" and "Nineteenth century studies about absinthism". DWL contributed the sections "Definition of pre-ban absinthe", "Pre-ban absinthe – a target for food adulteration", "Modern absinthe", "Modern studies about pharmacology and toxicology of thujone", "Toxicological Rehabilitation of Absinthe", "Present Impact" and revised the final draft. All authors read and approved the final manuscript.
